# Kidney autotransplantation as a treatment for resistant hypertension due to renal artery stenosis: A case report and review of the literature 

**DOI:** 10.5414/CNCS110565

**Published:** 2022-01-05

**Authors:** Tahira Scott, Sree Krishna Venuthurupalli

**Affiliations:** 1 Department of Nephrology, Princess Alexandra Hospital,; 2 School of Medicine, University of Queensland, Brisbane, and; 3 Department of Nephrology, West Moreton Health, Ipswich, Australia

**Keywords:** hypertension, renal artery stenosis, kidney autotransplant

## Abstract

Resistant hypertension is a common presentation of renal artery stenosis. Hypertension secondary to renal artery stenosis is typically managed with lifestyle and pharmacological interventions and less commonly with angioplasty or stenting, although exact treatment varies depending on the cause. In select cases refractory to these measures, kidney autotransplantation may be a valuable last-line approach. This case report demonstrates the successful use of kidney autotransplant for managing resistant hypertension in a young male with Takayasu’s arteritis and renal artery stenosis of a solitary kidney. We review the literature on the indications for kidney autotransplantation in renal artery stenosis, including the outcomes on blood pressure control and renal function and also the potential complications.

## Introduction 

Renal artery stenosis is a major cause of secondary hypertension in the United States [1]. Renal artery narrowing leads to compromised kidney blood supply and activation of the renin-angiotensin-aldosterone system with subsequent blood pressure elevation. The commonest cause of renal artery stenosis is atherosclerotic renovascular disease, followed by fibromuscular dysplasia and vasculitides such as Takayasu’s arteritis. Renovascular hypertension often presents as resistant hypertension. Resistant hypertension from renovascular disease is typically managed with lifestyle and pharmacological interventions and less commonly with angioplasty or stenting, although exact treatment varies depending on the cause. In select cases refractory to these measures, kidney autotransplantation may be a valuable last-line approach [2]. 

We present a case of a patient with resistant hypertension from Takayasu’s arteritis and bilateral renal artery stenosis who underwent kidney autotransplantation as a last-line therapy. 

## Case report 

A 27-year-old Caucasian male presented to the emergency department with headaches and a blood pressure of 225/140 mmHg. His past medical history was significant for Takayasu’s arteritis complicated by bilateral renal artery stenosis and resistant hypertension. He had had a left radical nephrectomy at 5 years of age due to renal atrophy and hypertension, and open aortorenal vascular grafting of the right kidney at 10 years of age for severe stenosis, which required a revision at 14 years of age due to graft thrombosis. The disease-modifying treatment of Takayasu’s arteritis in childhood was unknown, however, his disease was quiescent by adolescence, and he no longer required immunosuppression. Hypertension was identified in childhood, and he had been prescribed metoprolol and amlodipine since diagnosis. He underwent regular outpatient review with a nephrologist for blood pressure management in the context of a solitary kidney. The patient reported his blood pressure readings to be good, although the outpatient blood pressure records were unable to be obtained for correlation. 

On examination, the patient had no encephalopathy or focal neurological signs. Fundoscopy revealed moderate hypertensive changes. There was a displaced apex beat and a fourth heart sound indicative of cardiomegaly and diastolic dysfunction. A renal bruit was noted in the right lower quadrant. Features of left ventricular hypertrophy were noted on electrocardiogram, and transthoracic echocardiography confirmed end-organ damage with a severely enlarged left ventricular mass index of 170 g/m^2^. His metabolic parameters at the time were normal, with a potassium 4.2 mmol/L, bicarbonate 23 mmol/L, and creatinine 79 µmol/L, ESR 4 mm/h, and albuminuria with a urine albumin/creatinine ratio 23 g/mol creatinine. 

**Figure 1:**
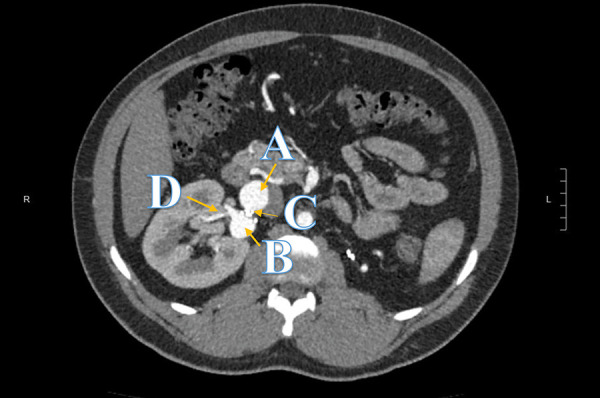
Computer tomography angiogram displaying on transverse view the right solitary kidney with two aneurysms (A & B), mid graft stenosis (C), and hilar stenosis (D).

The patient was discharged for outpatient follow-up. His medications were titrated in the community to amlodipine 20 mg daily, hydrochlorothiazide 25 mg daily, topical glyceryl trinitrate 10 mg/24 h, metoprolol tartrate 200 mg twice a day, prazosin 10 mg twice a day, and minoxidil 5 mg twice a day. Given sub-optimal blood pressure control, the patient subsequently underwent digital subtraction angiography, which showed moderate mid-graft stenosis of a right aortorenal bypass, large false aneurysms of the venous graft anastomoses, and hilar renal artery stenosis (Figure 1). Although the complex vascular anatomy was deemed unamenable to percutaneous intervention, conservative management placed the patient at risk of uncontrolled hypertension and further end-organ complications or aneurysm rupture. It was decided after multidisciplinary consultation that nephrectomy followed by retransplanting of his native kidney with anastomosis to the iliac vessels was a feasible final treatment option. 

The kidney autotransplant surgery was a complex, lengthy procedure taking 12 hours. The native right kidney was nephrectomized followed by back table preparation and reconstruction using cryopreserved cadaveric vessels. This was followed by transplantation into the lower right pelvis. The cold ischemic time was 210 minutes, and warm ischemic time was 15 minutes with urine produced immediately after unclamping. The patient developed anuria after wound closure requiring reopening at which a dusky kidney and kinked renal artery were identified and corrected. 

Post-operatively, the patient sustained an oliguric acute kidney injury (AKI) with a creatinine peak of 273 µmol/L on day 3 post-surgery. The patient was fluid overloaded with a weight gain of 10 kg by day 1 post-operatively. Intravenous furosemide was prescribed and oral antihypertensives titrated to maintain a systolic blood pressure below 160 mmHg. No dialysis was required, and upon discharge on day 7 post-operatively, the creatinine was 109 µmol/L. Discharge medications were amlodipine 10 mg daily, metoprolol tartrate 100 mg twice a day, prazosin 5 mg twice a day, and aspirin 100 mg daily. 

At 6 weeks’ follow-up, the patient’s creatinine had improved to 59 µmol/L, and his blood pressure was well controlled at 110/70 mmHg on amlodipine 10 mg daily monotherapy. 

## Discussion 

Kidney autotransplantation was first performed by Dr James Hardy, a urologist, in the United States in 1963 [3]. It was undertaken to salvage a single functioning kidney in a patient with a past traumatic ureteric transection, which after repair developed an ureteroureteric anastomotic stricture. The kidney was excised and reimplanted into the pelvis with attachment to the external iliac vessels [3], and the outcome was successful. For context, the first living donor allogeneic kidney transplantation was performed in 1954 [4]. 

Kidney autotransplantation is an uncommon procedure and is reserved as a last-line measure in highly selected circumstances. The commonest indications in descending order are ureteric stricture, tumors of the kidney, resistant hypertension secondary to renal artery stenosis, and chronic loin pain hematuria syndrome [5]. Of patients undergoing kidney autotransplantation for resistant hypertension, the most frequent underlying etiologies are fibromuscular dysplasia (50%), Takayasu’s arteritis (38%), and atherosclerotic renovascular disease (12%) [6]. 

Our paper describes a patient with the typical lesion of Takayasu’s arteritis, with occlusive obliteration and aneurysmal degeneration of the renal arteries. Renal artery stenosis and aneurysm formation is common in Takayasu’s arteritis and can result in kidney impairment, resistant hypertension, and risk of vessel rupture [7]. Operative management is frequently required once conservative treatments fail. Patients are usually subjected to angioplasty, stenting, aortorenal bypass, or nephrectomy, with kidney autotransplantation being the rarest intervention [7]. For complex vascular lesions, such as with our case, anatomical indications for autotransplantation include dual arterial lesions at the main artery and hilar branches and aneurysms and tight ostial lesions not amendable to angioplasty or stenting [8]. 

Several observational studies have reported that kidney autotransplantation appears to be an effective treatment for resistant hypertension, with 86 – 91% of patients achieving normotension following surgery with a mean follow-up of 4.3 – 5 years [9] [6]. A Chinese cohort study reviewed the mean pre and post blood pressure readings and number of anti-hypertensive agents pre and post kidney autotransplant in a small cohort undergoing the surgery for renovascular hypertension. They found a mean pre-operative systolic blood pressure of 204 mmHg compared with a mean systolic blood pressure of 129 mmHg at 3.4 years after surgery. They also reported a reduction in antihypertensive agents from 3.4 to 0.2 before and after surgery, respectively, to achieve an adequate blood pressure reading < 140/90 mmHg [10]. Another group in Turkey found the mean blood pressure reductions to be 168 mmHg systolic to 128 mmHg with an average follow-up of 9.8 years post kidney autotransplant of the kidney [11]. 

Data also shows that the surgery is able to preserve kidney function in 88 – 95% of cases [6, 9, 12]. Long-term data from Tran et al. [12] followed a cohort of 52 patients for 73.5 months post kidney autotransplant. Their group showed that 90% of cases had normal kidney perfusion on renal Doppler and remained free of dialysis with a median baseline creatinine at last follow-up of 88.42 µmol/L. Within the cohort, 1 patient became dialysis dependent due to declining graft function of his solitary kidney, and 4 others developed progressive chronic kidney disease due to vascular and stricture complications necessitating kidney autotransplant nephrectomy [12]. 

Kidney autotransplantation is often performed in younger patients with the mean age at the time of surgery being 41 – 48 years of age and are more often performed in women [12, 13]. The kidney autotransplant is a complex operation. The mean surgery time is more than 360 minutes and is prolonged if vascular repair, or preparation is required on the back table. The associated cold ischemic time – being the period during which the kidney vessels have been clamped and perfusion is absent with the graft being placed in ice – ranges from ~ 51 – 209 minutes [5, 13]. This is shorter than is reported for living donor kidney transplantation, for which cold ischemic time exceeds 120 minutes in greater than 80% of cases [14]. 

Meticulous peri-operative planning between both the nephrologist and transplant surgeon is required. The nephrologist’s role is to optimize the patient’s medical comorbidities and particularly to monitor volume status and blood pressure control. Ideal blood pressure targets in this context are uncertain, however, limited literature suggests aiming for a systolic blood pressure below 160 mmHg [15], as this appears in a retrospective cohort study to be associated with reduced post-operative morbidity and mortality [16]. There is also a risk of AKI, and similar to allogenic transplantation, a prolonged cold ischemic time is predictive of delayed graft function [14]. 

Additional post-operative considerations are immunosuppression and anti-platelets. Often, autologous vessels are used for autotransplantation [6], however, in this case, cadaveric vessel allograft was used due to the extent of the patient’s damaged native vessels. Limited experimental animal studies have suggested that immune-suppressing treatment, such as corticosteroids or calcineurin inhibitors, confer little benefit [17, 18], and it is postulated that cryopreservation reduces vessel allograft antigenicity [19]. Immunosuppression is therefore not recommended for cadaveric vessels [18]. An antiplatelet agent, generally aspirin, is recommended for a minimum of 6 months [20] to prevent vessel graft thrombosis (11). Albeit, the duration of anti-platelets is often determined by the protocol of the transplant center, similar to allogenic kidney transplants [21]. 

Although overall uncommon, important post-surgical complications include kidney failure requiring dialysis, an increased risk of subsequent chronic kidney disease, vessel thrombosis, bleeding, and urine leak. In the post-operative setting of a rising creatinine or oliguria, a renal doppler ultrasound is the first-line test for ruling out these complications. However, if there is heightened suspicion of vascular thrombosis, a computer tomography is needed. In the largest cohort study to date, the most common complication was vessel thrombosis with 3.6% of kidneys being lost due to thrombus in the early transplant period [22]. Lastly, although it is rare, there has been a reported case of recurrent Takayau’s arteritis in an iliac graft leading to vascular compromise [23]. Across all indications for kidney autotransplant, 30-day mortality rates remain low, ~ 4% [22, 24]. 

## Conclusion 

In conclusion, this case demonstrates the success of a kidney autotransplant as a treatment for resistant hypertension in complex vascular lesions that lead to renovascular hypertension. It serves as a reminder to nephrologists that in those with renovascular hypertension from more infrequent etiologies, patients may require surgical referral for intervention other than angiography for optimizing blood pressure control to avoid hypertensive end-organ complications and achieve nephron preservation. 

## Funding 

The authors have no financial or other support to acknowledge. 

## Conflict of interest 

There is no conflict of interest noted. 

## References

[1] Charles L Triscott J Dobbs B Secondary hypertension: Discovering the underlying cause. Am Fam Physician. 2017; 96: 453–461. 29094913

[2] Azhar B Patel S Chadha P Hakim N Indications for renal autotransplant: an overview. Exp Clin Transplant. 2015; 13: 109–114. 25871361

[3] Hardy JD High ureteral injuries. Management by autotransplantation of the kidney. JAMA. 1963; 184: 97–101. 13960761 10.1001/jama.1963.03700150051008

[4] Barker CF Markmann JF Historical overview of transplantation. Cold Spring Harb Perspect Med. 2013; 3: a014977. 23545575 10.1101/cshperspect.a014977 PMC3684003

[5] Ruiz M Hevia V Fabuel J-J Fernández A-A Gómez V Burgos F-J Kidney autotransplantation: long-term outcomes and complications. Experience in a tertiary hospital and literature review. Int Urol Nephrol. 2017; 49: 1929–1935. 28828690 10.1007/s11255-017-1680-1

[6] Chiche L Kieffer E Sabatier J Colau A Koskas F Bahnini A Renal autotransplantation for vascular disease: late outcome according to etiology. J Vasc Surg. 2003; 37: 353–361. 12563206 10.1067/mva.2003.84

[7] Mwipatayi BP Jeffery PC Beningfield SJ Matley PJ Naidoo NG Kalla AA Kahn D Takayasu arteritis: clinical features and management: report of 272 cases. ANZ J Surg. 2005; 75: 110–117. 15777385 10.1111/j.1445-2197.2005.03312.x

[8] Mhaske SM Patil B Patwardhan SK Gopalakrishnan G Shelke UR Pamecha YG Outcome following renal autotransplantation in renal artery stenosis. Urol Ann. 2019; 11: 46–52. 30787570 10.4103/UA.UA_39_18 PMC6362799

[9] Brekke IB Sødal G Jakobsen A Bentdal O Pfeffer P Albrechtsen D Flatmark A Fibro-muscular renal artery disease treated by extracorporeal vascular reconstruction and renal autotransplantation: short- and long-term results. Eur J Vasc Surg. 1992; 6: 471–476. 1397339 10.1016/s0950-821x(05)80619-x

[10] Li FD Ji ZG Liu CW Shao J Xie Y Zheng YH Orthotopic renal autotransplantation for young-onset and medical treatment-requiring complex renovascular hypertension. J Renin Angiotensin Aldosterone Syst. 2018; 19: 1470320318789861. 30129810 10.1177/1470320318789861 PMC6104217

[11] Sevmis S Karakayali H Boyvat F Aytekin C Haberal M Renal autotransplantation for the treatment of complex renovascular hypertension. Transplant Proc. 2006; 38: 3412–3415. 17175289 10.1016/j.transproceed.2006.10.143

[12] Tran G Ramaswamy K Chi T Meng M Freise C Stoller ML Laparoscopic nephrectomy with autotransplantation: safety, efficacy and long-term durability. J Urol. 2015; 194: 738–743. 25801764 10.1016/j.juro.2015.03.089 PMC4966611

[13] Bourgi A Aoun R Ayoub E Moukarzel M Experience with Renal Autotransplantation: Typical and Atypical Indications. Adv Urol. 2018; 2018: 3404587. 29780413 10.1155/2018/3404587 PMC5892291

[14] Simpkins CE Montgomery RA Hawxby AM Locke JE Gentry SE Warren DS Segev DL Cold ischemia time and allograft outcomes in live donor renal transplantation: is live donor organ transport feasible? Am J Transplant. 2007; 7: 99–107. 17227561 10.1111/j.1600-6143.2006.01597.x

[15] Meng L Yu W Wang T Zhang L Heerdt PM Gelb AW Blood pressure targets in perioperative care. Hypertension. 2018; 72: 806–817. 30354725 10.1161/HYPERTENSIONAHA.118.11688

[16] Reich DL Bennett-Guerrero E Bodian CA Hossain S Winfree W Krol M Intraoperative tachycardia and hypertension are independently associated with adverse outcome in noncardiac surgery of long duration. Anesth Analg. 2002; 95: 273–277. 12145033 10.1097/00000539-200208000-00003

[17] Kieffer E Gomes D Chiche L Fléron MH Koskas F Bahnini A Allograft replacement for infrarenal aortic graft infection: early and late results in 179 patients. J Vasc Surg. 2004; 39: 1009–1017. 15111853 10.1016/j.jvs.2003.12.040

[18] Chakfé N Diener H Lejay A Assadian O Berard X Caillon J Fourneau I Glaudemans AWJM Koncar I Lindholt J Melissano G Saleem BR Senneville E Slart RHJA Szeberin Z Venermo M Vermassen F Wyss TR de Borst GJ Bastos Gonçalves F Editor’s Choice - European Society for Vascular Surgery (ESVS) 2020 Clinical Practice Guidelines on the Management of Vascular Graft and Endograft Infections. Eur J Vasc Endovasc Surg. 2020; 59: 339–384. 32035742 10.1016/j.ejvs.2019.10.016

[19] Miller VM Bergman RT Gloviczki P Brockbank KGM Cryopreserved venous allografts: effects of immunosuppression and antiplatelet therapy on patency and function. J Vasc Surg. 1993; 18: 216–226. 8350430

[20] Hess CN Norgren L Ansel GM Capell WH Fletcher JP Fowkes FGR Gottsäter A Hitos K Jaff MR Nordanstig J Hiatt WR A structured review of antithrombotic therapy in peripheral artery disease with a focus on revascularization: A TASC (InterSociety Consensus for the Management of Peripheral Artery Disease) Initiative. Circulation. 2017; 135: 2534–2555. 28630267 10.1161/CIRCULATIONAHA.117.024469

[21] Robertson AJ Nargund V Gray DWR Morris PJ Low dose aspirin as prophylaxis against renal-vein thrombosis in renal-transplant recipients. Nephrol Dial Transplant. 2000; 15: 1865–1868. 11071979 10.1093/ndt/15.11.1865

[22] Flatmark A Albrechtsen D Sødal G Bondevik H Jakobsen A Brekke IB Renal autotransplantation. World J Surg. 1989; 13: 206–209, discussion 210.. 2658368 10.1007/BF01658401

[23] Robbs JV Abdool-Carrim ATO Kadwa AM Arterial reconstruction for non-specific arteritis (Takayasu’s disease): medium to long term results. Eur J Vasc Surg. 1994; 8: 401–407. 7916304 10.1016/s0950-821x(05)80957-0

[24] Vrakas G Sullivan M Current review of renal autotransplantation in the UK. Curr Urol Rep. 2020; 21: 33. 32666391 10.1007/s11934-020-00986-z PMC7360532

